# The Role of Opportunity to Learn on Student Mathematics Anxiety, Problem-Solving Performance, and Mathematics Performance

**DOI:** 10.3389/fpsyg.2022.829032

**Published:** 2022-02-17

**Authors:** Siwen Guo, Shanhui Liao

**Affiliations:** Department of Psychology, Renmin University of China, Beijing, China

**Keywords:** opportunity to learn (OTL), content coverage, mathematics anxiety, problem-solving, mathematics performance

## Abstract

This study examined the effects of opportunity to learn (OTL) or the content coverage in mathematics on student mathematics anxiety, problem-solving performance, and mathematics performance. The pathways examining the influences of OTL on student problem-solving performance and mathematics performance *via* mathematics anxiety were also tested. A sample of 1,676 students from Shanghai-China, and a sample of 1,511 students from the United States who participated in the Programme for International Student Assessment (PISA) 2012 were used for the analyses. The results from multilevel models and path models supported our hypotheses that OTL not only showed significant direct effects on student mathematics anxiety, problem-solving performance, and mathematics performance, but also presented indirect effects on student problem-solving performance and mathematics performance *via* mathematics anxiety in both Shanghai-China and United States, controlling for student gender, grade, and socioeconomic status. The practical implications of the current results were also discussed.

## Introduction

Academic anxiety is referred to as the students’ negative feelings of fears, anxieties, tensions, and worries in the academic context (Pekrun, 2006). A general negative relationship between academic anxiety and students’ cognitive and academic performance is documented in the literature (Pekrun et al., 2002; Ardasheva et al., 2018; Ramirez et al., 2019). Mathematics is found as one of those specific academic domains/subjects which trigger greater anxiety than others. As one of the core subjects throughout K-12 schooling across the world, mathematics not only provides content knowledge and tools for students to understand and adapt to the changing world, but also help to cultivate their cognitive skills, such as critical thinking, reasoning, and problem-solving skills, for their future successes in dealing with the complicated situations. A growing number of studies are paying attention to the mathematics anxiety. Based on previous research, students that suffer from mathematics anxiety generally perform worse in mathematics compared to others controlling their general mental abilities and mathematics abilities (Pajares and Kranzler, 1995; Wu et al., 2017; Schaeffer et al., 2018; Zhang et al., 2018; Donolato et al., 2020; Maldonado Moscoso et al., 2020). These students will also avoid mathematics-related activities even when they understand that mathematics is an essential course and provides necessary skills for their future career paths (Hasty et al., 2021; Huang et al., 2019; Meece et al., 1990; Krinzinger et al., 2009; Li et al., 2021).

Opportunity to learn (OTL) in mathematics is a key factor to understanding the effect of schooling. Previous research showed a significant and positive impact of OTL on student achievement, regardless of the students’ parental education level and income, and prior test scores (Cogan et al., 2001; Lleras, 2008; Schmidt et al., 2015). From the control-value theory of achievement emotions (Pekrun et al., 2002; Pekrun, 2006), mathematics anxiety may be related to many factors, like situational perceptions, cognitive appraisals, physiological processes, feedbacks, etc. When students feel a low control of the mathematics-related situations, and the outcomes under these situations are of high value to them, they may suffer from mathematics anxieties. The control-value theory indicates that by raising their senses of self-control, students may experience less mathematics anxiety. When students are exposed to a better content coverage in mathematics, or they are provided with better OTL in mathematics, it is reasonable to assume that students will feel more confident and have a greater sense of control in mathematics-related activities, and their mathematics anxiety will be released, and they will be more likely to achieve better mathematics outcomes. Thus, the current study aimed at exploring the direct effect of OTL on students’ mathematics anxiety, and its indirect effects on mathematics performance and mathematics-related cognitive skills *via* mathematics anxiety.

## Literature Review

### Mathematics Anxiety

Mathematics anxiety describes students’ uncomfortable feelings when they deal with numbers, use mathematical concepts, learn mathematical knowledge, or solve mathematical problems (Carey et al., 2016). It is defined as the feelings of tension, helplessness, and mental disorganization due to manipulation of numbers and solving mathematical problems (Ma, 1999; Sorvo et al., 2017). In addition to the psychological feelings, students who experience mathematics anxiety may also have physical reactions, like sweating and pain. Previous literature documented the negative effects of mathematics anxiety on student academic motivations, attitudes toward mathematics, mathematics achievement, and future mathematical learnings (Gierl and Bisanz, 1995; Luo et al., 2014; Wu et al., 2014, 2017; Carey et al., 2016; Ramirez et al., 2018; Namkung et al., 2019; Donolato et al., 2020; Pantoja et al., 2020; Westfall et al., 2020; Barroso et al., 2021). Recently, Barroso et al. (2021) conducted a meta-analysis of the relationship between mathematics anxiety and student mathematics achievement across the studies from 1992 to 2018, and found a general negative correlation, i.e., *r* =–0.28, between them. The relationship between mathematics anxiety and mathematics achievement was moderated by student gender, race, mathematical ability, mathematics anxiety scale, mathematics assessment type, etc. Zhang et al. (2019) and Namkung et al. (2019) also found a negative and significant relationship between mathematics anxiety and mathematics achievement in their meta-analyses.

Researchers from different perspectives have given plenty of efforts to explain the adverse effects of mathematics anxiety on mathematics performance. The attentional control theory is the most influential one. It claims that mathematics anxiety impairs the efficient functioning of the goal-directed attentional system and increases one’s attention to threat-related stimuli. By affecting the inhibition and shifting functioning, mathematics anxiety may lead to one’s poor mathematics performance for the deficient recruitment of attentional control resources (Eysenck and Calvo, 1992; Eysenck et al., 2007; Eysenck and Derakshan, 2011). The attentional control theory helps to explain the difference in mathematics performance between high- and low-anxious individuals with similar mathematics abilities under stressful conditions. Empirical research provided some evidence for the attentional control theory on the mechanisms of mathematics anxiety (Passolunghi et al., 2016; Justicia-Galiano et al., 2017; Novak and Tassell, 2017; Skagerlund et al., 2019; Orbach et al., 2020; Szczygiel, 2021). Some other researchers further suggested that the negative relationship between mathematics anxiety and mathematics performance was related to mathematics avoidance behaviors. To be specific, the students with mathematics anxiety may avoid mathematics-related situations or they may choose to reduce their exposure to mathematics (Carey et al., 2016; Ramirez et al., 2018). Choe et al. (2019) used a novel effort-based decision-making paradigm and experimentally demonstrated a significant association between mathematics anxiety and mathematics avoidance.

Not only on mathematics performance, but mathematics anxiety was also found with a negative effect on student mathematics-related cognitive skills (Wu et al., 2017; Maloney and Retanal, 2020), especially on problem-solving skills (Pajares and Kranzler, 1995; Pajares, 1996; Zhang et al., 2018; Ozcan and Gumus, 2019; Passolunghi et al., 2019; Zhou et al., 2020). The development of problem-solving skills is considered mainly related to the mathematics domain. It requires one’s basic mathematical content knowledge and skills and calls for cognitive skills from students to grab the relevant information from the problem situations and figure out the possible solutions. Compared to simple mathematics tasks, it is highly likely to observe mathematics anxiety when students were involved in complex tasks requiring problem-solving skills (Wu et al., 2012; Ching, 2017). Previous research found that high mathematics anxiety disrupted students’ working memory resources and impeded their problem-solving performance (Beilock, 2008; Alamolhodaei, 2009; Buelow and Frakey, 2013; Ramirez et al., 2013, 2016; Trezise and Reeve, 2014; Zhang et al., 2018; Liu et al., 2019).

### Opportunity to Learn and Mathematics Anxiety

To relieve mathematics anxiety and alleviate its effects on student mathematics performance and problem-solving performance, researchers have explored the effects of students’ own characteristics and their parental cognitive, affective, and behavioral factors on student mathematics anxiety (Meece et al., 1990; Pajares and Kranzler, 1995; Hoffman, 2010; Gunderson et al., 2012; Maloney et al., 2015; Luo et al., 2016; Macmull and Ashkenazi, 2019; Sun et al., 2020; Sagkal and Sonmez, 2021), while little attention has been given on the daily teaching and instructional factors for remediating mathematics anxiety in students.

The literature in mathematics education shows the importance of OTL in mathematics for student learning. According to Carroll’s (1963) model, the coverage, depth, and rigor of mathematics content should be planned carefully based on students’ levels of aptitudes, skills, and knowledge, to promote their mathematics learning. To be specific, the instruction content should be clearly specified, the instructional materials should be well prepared and sequenced, and an adequate contact time with learning materials should be given for students (Carroll, 1963). In the mathematics curriculum literature, researchers from different perspectives use different indicators to measure OTL or learning opportunity. The types of courses (Riegle-Crumb and Grodsky, 2010), different educational tracks or programs (Cogan et al., 2001; Mickelson, 2001), different types of textbooks from different publishers (Shield and Dole, 2013), different content organizations (Grouws et al., 2013; Tar et al., 2013), content coverage or content exposure in mathematics (Cogan et al., 2019), etc. have been used. The indicators on curricular differentiation (e.g., types of courses, different educational tracks or programs, different types of textbooks from different publishers, and different content organizations) reflect some parts of the OTL, while they are too imprecise to identify students’ content exposure in mathematics. Although using different OTL indicators, the positive effects of OTL on student mathematics performance have been documented in these previous studies.

The results from the Programme for International Student Assessment (PISA) 2012 showed that the more formal education in mathematics students received, the better mathematics performance they got (OECD, 2014c). The content coverage in mathematics was statistically significantly related to student mathematics performance in all participating countries in PISA 2012 (Cogan et al., 2019). Significant and positive relationships between content coverage in mathematics and student achievement were found for K-12 students by different studies, controlling for students’ parental education level and family income, students’ own prior grade or test score, and curriculum implementation measures (Cogan et al., 2001; Byrnes and Miller, 2007; Lleras, 2008; Byrnes and Wasik, 2009; Reeves, 2012; Albano and Rodriguez, 2013; Grouws et al., 2013; Ottmar et al., 2014; Schmidt et al., 2015; Santibanez and Fagioli, 2016). Even for the low-achieving and low-income students, more rigorous content coverage in mathematics also contributed to higher mathematics achievement (Gamoran et al., 1997). A measure of OTL with seven items on the exposure to mathematics coursework, mathematics classroom experiences, and mathematics homework, also explained a significant variation in the ethnic gap in mathematics achievement (Byrnes, 2003). In addition, by using the advanced mathematics courses taking as a measure of OTL, the effect of OTL on mathematics performance is found to be highly likely continuous and cumulative, and will affect student mathematics development in the long run (Muller et al., 2010; Riegle-Crumb and Grodsky, 2010).

Opportunity to learn is not only critical for students to achieve good mathematics performance but also necessary for them to possess problem-solving skills. Lee and Chen (2009) conducted an experiment and found that students with high prior knowledge in pattern reasoning outperformed the low prior-knowledge group in non-routine problem-solving tasks. This result indicated that improving the prior knowledge might be the first step for teaching and learning non-routine mathematical problem-solving. Without a good OTL in mathematics, students will not get a high “prior content knowledge”. Although rare, the role of OTL in mathematics on student problem-solving performance has been studied. Grouws et al. (2013) found students who took different mathematics curricula with different content organizations had significantly different mathematics performances as well as different problem-solving performances. Tar et al. (2013) used the percentage of textbook lessons taught as a measure of OTL and found both OTL and content organizational structures showed significant effects on student mathematics performance and problem-solving performance.

The control-value theory throws light on the role of OTL in easing students’ mathematics anxiety, which states that students’ self-related and situational appraisals are the important determinants of their achievement emotions (Pekrun et al., 2002; Pekrun, 2006). When students value mathematics as important, but they cannot control mathematics-related activities, they will feel anxious. Based on the control-value theory, raising the quality of instruction may help to increase students’ sense of control, as well as ease their anxiety toward mathematics. Better coverage of mathematics content may be one of the important instructional strategies to alleviate students’ mathematics anxiety.

Empirical studies in education and psychology provided some evidence for the potentially beneficial effect of OTL on student mathematics anxiety. Ching et al. (2020) showed students’ prior quantitative reasoning and number knowledge had significant effects on their later mathematics anxiety controlling for their gender, mothers’ educational levels, and general anxiety level in a cross-lagged panel analysis; while student mathematics anxiety measured on Time 1 did not have any significant effect on students quantitative reasoning and number knowledge on Time 2. In other words, the poorer knowledge in quantitative reasoning and number students have, the more anxious they may feel in mathematics-related activities. Tomasetto et al. (2021) also showed student mathematics anxiety was negatively related to their initial level of mathematics knowledge. It is the content coverage in school daily instruction from which most students mainly learn mathematics. Frenzel et al. (2007) found the perceived quality of mathematics instruction (i.e., including clarity and structure) had a significant and negative effect on student mathematics anxiety. The interventions which improved student mathematics knowledge and skills were also shown as an effective way to reduce student mathematics anxiety. By participating in an 8-week intensive and well-validated mathematics-tutoring program, which combined conceptual instruction with speeded retrieval of arithmetical facts, the high mathematics-anxious students showed a reduction in their mathematics anxiety (Supekar et al., 2015). Supekar et al. (2015) thought the tutoring program helped to make these students feel more in control of the situation involving mathematical problem-solving, and thereby alleviated their mathematics anxiety. Passolunghi et al. (2020) conducted two different training programs, i.e., mathematics anxiety training and mathematics strategy training, on two groups of four-grade students. In each training program, a 60-min session was administered weekly over a period of 8 weeks. The mathematics anxiety training program only showed an effect on student mathematics anxiety, while the mathematics strategy training program which used supplementary exercises to enhance students’ calculation strategies showed a reduction in student mathematics anxiety as well as an improvement in student achievement (Passolunghi et al., 2020). They thought the students in the mathematics strategy training program might “develop a feeling of self-control in situations involving mathematical tasks, thereby reducing their mathematics anxiety (Passolunghi et al., 2020, p. 8),” and students were also influenced by the program in different mathematics abilities, such as numerical knowledge and word problem-solving abilities.

## The Current Study

The literature showed that, with better OTL in mathematics from school mathematics lessons or tutoring programs, students would feel more self-control and confidence in mathematics, which would alleviate their mathematics anxiety (Jansen et al., 2013). The reduction in mathematics anxiety would further bring benefits to student mathematics achievement as well as their problem-solving skills, in addition to the direct effects brought by OTL.

As discussed in the literature review, various OTL measures have been used in the previous studies. These indicators on curricular differentiation reflect some parts of the OTL in Carroll’s (1963) model, while they are too imprecise to identify students’ content exposure in mathematics. Cogan et al. (2001) found the mathematics classes with the same course title and taught in the same school could offer different content to their students. In this study, rather than using the indicators of curriculum differentiation, the OTL index from PISA 2012 is used, which reflects what mathematics content students are exposed to in their daily mathematics lessons (OECD, 2014c). It is measured as the students’ responses on “how much they are familiar with or heard of that topic.” The topics in this scale cover the mathematics content that is typically taught in grades four to eleven. Given the sequencing nature of mathematics content and fixed instructional time for mathematics in each school year, the resulting index in fact indicates the amount of content exposure and the degree of emphasis on the necessary, important, and advanced topics in mathematics. That is to say, the OTL measure in the current study reflects the coverage, depth, and rigor of mathematics content students encounter. This content coverage in mathematics was argued as a more precise indicator of OTL and was shown with a significantly positive effect on student mathematics performance in the previous studies (Schmidt et al., 2015, 2021; Cogan et al., 2019).

**FIGURE 1:**
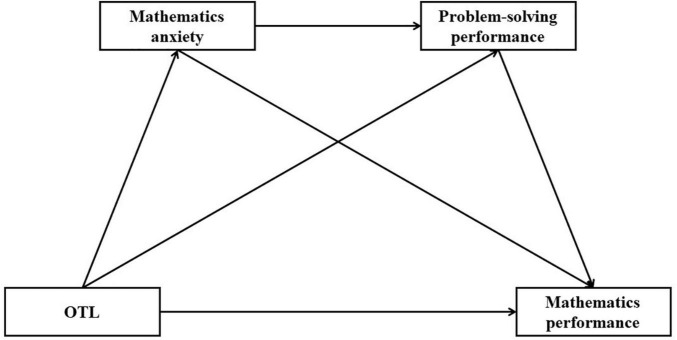
Theoretical model.

Thus, the current study aimed at exploring the direct and indirect effects of content coverage in mathematics or OTL *via* mathematics anxiety on student mathematics performance and problem-solving performance (shown in Figure 1). As problem-solving skills generally showed direct effects on student mathematics performance (Vista, 2016), and problem-solving skills might work as a mediator between mathematics anxiety and mathematics performance (Ramirez et al., 2016), the effects of OTL and mathematics anxiety on student mathematics performance were assumed to be mediated by problem-solving performance in this study.

## Materials and Methods

### Data

Program for International Student Assessment (PISA) is an international standardized assessment that measures how well-prepared 15-year-old students are for their future life (OECD, 2014c,d). In each round of assessment, student mathematics, reading, and science literacies are measured with a major subject measured with greater precision. In PISA 2012, the major subject is mathematics. Not only student mathematics performance, but also student problem-solving performance was tested in this round of assessments. Paper-based tests were used to assess students’ mathematics literacy, with an additional optional computer-based assessment of mathematics, reading, and problem-solving. Students also needed to answer a contextual questionnaire about themselves, their homes, and their school and learning experiences (OECD, 2014c). The content coverage in mathematics lessons as well as students’ attitudes toward mathematics were measured in student surveys (OECD, 2014c,d). This provided the possibilities for the current study to examine the effects of OTL on student mathematics anxiety, problem-solving skills, and mathematics achievement in a detailed manner. Previous research showed the differences in mathematics anxiety structures and effects on student achievement between eastern and western countries (Lee, 2009; Morony et al., 2013; Zhu and Meyer, 2020). To clarify the indirect effects of OTL on student mathematics performance and problem-solving performance *via* mathematics anxiety, Shanghai-China and United States samples were selected for comparison.

A stratified two-stage sampling design was used in each country/region (i.e., 34 OECD countries/regions and 31 partner countries/regions) in PISA 2012, where schools were sampled using probability proportional to size sampling (PPS), and students were sampled with equal probabilities within sampled schools. There were about 150 schools drawn from each country/region, with around 30 sampled students within each sampled school (OECD, 2014c,d). PISA 2012 used a random rotated block design for student surveys. Only one-third of students answered both OTL and mathematics anxiety items, which left about 10 students in each school. After deleting the cases with missing data in the analyses, there were 1,676 students in 155 schools from Shanghai-China, and 1,511 students in 160 schools from the United States in the current study.

### Measures

#### Mathematics Performance

In PISA, mathematics proficiency is defined as “the capacity of individuals to formulate, employ, and interpret math in a variety of contexts (OECD, 2014c, p. 25).” The mathematics test items are used to assess the “capacities of individuals to reason mathematically and use mathematical concepts, procedures, facts, and tools to describe, explain, and predict phenomena (OECD, 2014c, p. 25).” The test items are a mixture of multiple-choice items and questions on four mathematical content domains, which are quantity, uncertainty and data, change and relationships, and space and shape (OECD, 2014c). For the paper-based assessment, a total of 110 cognitive items is used with approximately 270 min of testing time in PISA 2012 (OECD, 2014d). The mean mathematics performance across OECD countries is 494 and the standard deviation is 92.

#### Problem-Solving Performance

Problem-solving performance reflects an “individual’s capacity to engage in cognitive processing to understand and resolve problem situations where a method of solution is not immediately obvious. It includes the willingness to engage with such situations to achieve one’s potential as a constructive and reflective citizen (OECD, 2014a, p. 30).” A major difference between problem-solving performance and mathematics performance is that the former requires students to perform non-routine analytic tasks, and they need to engage with the problem situations, monitor the effectiveness of their actions, as well as adjust to the feedback. “The problem-solving assessment in PISA 2012 focuses on students’ general reasoning skills, their ability to regulate problem-solving processes, and their willingness to do so, by confronting students with problems that do not require expert knowledge to solve (OECD, 2014a, p. 28).” Student problem-solving performance is assessed in the computer-based assessment in PISA 2012, which focuses on “students’ general reasoning skills, their ability to regulate problem-solving processes, and their willingness to do so, by confronting students with problems that do not require expert knowledge to solve.” The problem-solving assessment comprises 42 items which need 80 min of testing time in total. The mean problem-solving performance across OECD countries is 500 and the standard deviation is 100.

#### Opportunity to Learn

The OTL measure in the current study is constructed based on students’ exposure to formal mathematics on three scales. The first scale asks students about their familiarities with three algebra topics. The second scale asks students about their familiarities with four geometric topics. The response categories of these two scales vary from “never heard of it” which is scored as 0, to “knew it well” which is scored as 4. The third scale asks about their frequencies of being confronted with formal math problems, and the frequency categories “frequently,” “sometimes,” and “rarely” are coded equal to 1, and “never” is coded equally to 0. These topics in this scale cover the mathematics content that is typically taught at grades 4–11, which were selected by the mathematics experts from PISA. The OTL measure is a weighted score of all three of the elements of OTL described above, which ranges from 0 to 3. It reflects the coverage of the key topics and the degree of emphasis in daily mathematics instructions.

##### Mathematics Anxiety

Mathematics anxiety is referred to as the “feelings of helplessness and stress when dealing with mathematics (OECD, 2014b, p. 88).” The index of mathematics anxiety in PISA 2012 is constructed based on students’ responses about feelings of stress and helplessness when dealing with mathematics, with five items on the four-point Likert-type response scales. The response categories range from “strongly agree” to “strongly disagree”. The higher score on the index of mathematics anxiety, the higher the anxiety level the students have.

#### Control Variable

Except for the variables mentioned above, student socioeconomic background, grade, and gender are also considered in the analyses. Student grade is calculated as the relative grade level compared to the modal grade (i.e., which was given a value of 0) in PISA 2012. Student socioeconomic background is represented by the economic, social, and cultural status (ESCS) in PISA. ESCS is computed as a weighted score of students’ home possessions, parents’ occupations, and parents’ education levels. This variable has an average score of 0 and a standard deviation of 1 across OECD countries (OECD, 2014c,d). For gender, boys are coded as 1, and girls are coded as 0.

### Analysis

To examine the effects of OTL on student mathematics anxiety, problem-solving performance, and mathematics performance, two-level random intercept models were firstly conducted in United States and Shanghai-China. Then, a path analysis (as shown in Figure 1) was conducted to examine the direct effects of OTL on mathematics performance and problem-solving performance, as well as its indirect effects *via* mathematics anxiety. Student gender, grade, and ESCS were considered as covariates for OTL, mathematics anxiety, problem-solving performance, and mathematics performance in all models.

In light of PISA’s sampling design, the balanced repeated replication (BRR) method was used to estimate the standard errors for all estimates in the present study with BRR weights provided by PISA. Five plausible values were used in the analyses for student problem-solving performance and mathematics performance.

## Results

### Description

The means, standard deviations, and correlations between variables in the United States and Shanghai-China samples were presented in Table 1. On average, compared to the sample from the United States, students from Shanghai-China had more OTL, higher level of mathematics anxiety, better performance in the problem-solving test, and better performance in mathematics. The correlations among OTL, mathematics anxiety, problem-solving performance, and mathematics performance were significant in both the Shanghai-China sample and the United States sample. Student socioeconomic status, i.e., ESCS, was significantly correlated with all other variables in both samples. Student relative grade was significantly correlated with all other variables in Shanghai-China, but showed nonsignificant correlations with student gender and mathematics anxiety in the United States. Student gender was significantly correlated to mathematics anxiety and problem-solving performance in Shanghai-China, and significantly correlated to mathematics anxiety in the United States.

**TABLE 1:** Means (*M*), standard deviations (*SD*), and correlations among variables.

	1	2	3	4	5	6	7
**Shanghai-China sample (*n* = 1,676)**							
1. Grade							
2. Gender^a^	-0.10(0.02)***						
3. ESCS	0.19(0.03)***	-0.04(0.02)					
4. OTL	0.36(0.04)***	-0.06(0.03)	0.30(0.04)***				
5. Mathematics anxiety	0.06(0.02)**	-0.22(0.03)***	-0.08(0.02)***	-0.16(0.02)***			
6. Problem-solving performance	0.27(0.04)***	0.14(0.03)***	0.36(0.03)***	0.35(0.03)***	-0.26(0.03)***		
7. Mathematics performance	0.26(0.04)***	0.02(0.03)	0.39(0.03)***	0.45(0.03)***	-0.34(0.02)***	0.78(0.03)***	
*M*	-0.49(0.02)	0.48(0.01)	-0.34(0.04)	2.37(0.02)	2.40(0.02)	539.18(3.55)	615.85(3.54)
*SD*	0.64(0.02)	0.50(0.00)	0.95(0.02)	0.42(0.02)	0.68(0.01)	87.66(2.56)	99.36(2.67)
**United States sample (*n* = 1,511)**							
1. Grade							
2. Gender^a^	-0.06(0.03)						
3. ESCS	0.09(0.03)**	-0.01(0.03)					
4. OTL	0.29(0.03)***	-0.03(0.04)	0.32(0.03)***				
5. Mathematics anxiety	-0.05(0.03)	-0.13(0.02)***	-0.11(0.03)***	-0.30(0.03)***			
6. Problem-solving performance	0.22(0.03)***	0.02(0.03)	0.29(0.03)***	0.40(0.02)***	-0.36(0.02)***		
7. Mathematics performance	0.27(0.04)***	0.01(0.03)	0.35(0.03)***	0.53(0.02)***	-0.37(0.02)***	0.82(0.03)***	
*M*	0.09(0.02)	0.51(0.02)	0.23(0.04)	2.06(0.02)	2.25(0.02)	508.46(4.22)	488.01(3.60)
*SD*	0.54(0.02)	0.50(0.00)	0.96(0.02)	0.60(0.01)	0.75(0.01)	92.92(2.61)	88.84(1.90)

*Standard errors are reported in parentheses.*

*^a^Boy = 1; girl = 0.*

***p < 0.001.

**p < 0.01.

*p < 0.05.

### The Effects of OTL on Student Mathematics Anxiety, Problem-Solving Performance, and Mathematics Performance

To examine the effects of OTL on student mathematics anxiety, problem-solving performance, and mathematics performance, two-level random intercept models were built separately with mathematics anxiety, problem-solving performance, and mathematics performance as the outcomes. For each sample, the null model was first used to estimate the student-level and school-level variances of the outcome variable, as well as the intraclass correlation coefficients (ICC). Then, the effects of OTL were examined in each sample controlling for student gender, grade, and ESCS. The effects of mathematics anxiety on problem-solving performance and mathematics performance were explored, and student problem-solving performance was also included as a predictor of student mathematics performance in the models.

For student mathematics anxiety, the ICCs of anxiety were 2 and 2% in Shanghai-China and the United States, which suggested the nested structure of the data could be ignored. Regressions were used instead to examine the effect of OTL on student mathematics anxiety controlling for student characteristics (in Table 2). In general, girls presented a higher average level of mathematic anxiety. OTL showed significant negative effects on student mathematics anxiety in both Shanghai-China and the United States, which explained an additional 3 and 8% of the variance in mathematics anxiety in these two samples. With a high level of OTL, students tended to show less mathematics anxiety on average.

**TABLE 2:** Effects of OTL on student mathematics anxiety.

	Shanghai-China		United States	
	Model 1	Model 2	Model 1	Model 2
Intercept	2.54(0.02)***	3.38(0.11)***	2.37(0.03)***	3.14(0.09)***
Grade	0.06(0.02)**	0.12(0.02)***	–0.06(0.04)	0.05(0.05)
Sex	–0.29(0.04)***	–0.30(0.04)***	–0.19(0.04)***	–0.20(0.03)***
ESCS	–0.07(0.02)***	–0.04(0.02)	–0.09(0.02)***	–0.02(0.02)
OTL		–0.33(0.04)***		–0.38(0.04)***
*R* ^2^	0.06(0.01)***	0.09(0.01)***	0.03(0.01) **	0.11(0.02)***

*Standard errors are reported in parentheses.*

***p < 0.001.

**p < 0.01.

*p < 0.05.

For student problem-solving performance, the results from the two-level models are presented in Table 3. From the null models, the ICCs of problem-solving performance were 39% in Shanghai-China, and 28% in the United States, which indicated the necessity to use the multilevel modeling approach for both samples. Student OTL and mathematics anxiety, as well as student-level covariates, were included in Model 1 for both samples. These variables explained 15% of the student-level variance and 39% of the school-level variance in Shanghai-China, and 26% of the student-level variance and 26% of the school-level variance in the United States. Student grade and ESCS showed significant effects on student problem-solving performance in both Shanghai-China and United States. With higher relative grades and higher ESCS, students tended to achieve better performance in problem-solving tests controlling for other variables. Student gender showed a significant effect on student problem-solving performance in Shanghai-China, but not in the United States. Only in Shanghai-China, boys had a significantly higher average score in problem-solving than girls. In both Shanghai-China and United States, OTL showed significant and positive effects on student problem-solving performance (in Shanghai-China, β = 24.01,*p* < 0.01; in the United States, β = 30.59,*p* < 0.001), and mathematics anxiety showed significant and negative effects on student problem-solving performance (in Shanghai-China, β = −21.83,*p* < 0.001; in the United States, β = −33.77,*p* < 0.001) controlling for the effects of other variables. These results indicated that the students who were exposed to more content coverage and had lower levels of mathematics anxiety might achieve higher problem-solving performance on average.

**TABLE 3:** Effects of OTL on student problem-solving performance.

	Shanghai-China		United States	
	Null model	Model 1	Null model	Model 1
**Fixed effects**				
Intercept	537.27(1.20)***	538.13(20.20)***	507.49(1.22) ***	515.72(15.59)***
Grade		27.24(4.80)***		23.24(4.17)***
Sex		26.96(3.42)***		1.48(4.25)
ESCS		11.09(2.37)***		9.58(2.58)***
OTL		24.01(7.13) **		30.59(4.70)***
Mathematics anxiety		–21.83(2.94)***		–33.77(3.21)***
**Random effects**				
Student level σ^2^	4,700.99(226.45) ***	3,987.65(191.10)***	6,156.26(262.11) ***	4,548.34(217.56)***
School level τ_00_	2,995.68(228.26) ***	1,812.67(188.78)***	2,354.12(241.11) ***	1,741.71(202.44)***

*Standard errors are reported in parentheses.*

***p < 0.001.

**p < 0.01.

*p < 0.05.

For student mathematics performance, the results from the two-level models are presented in Table 4. From the null models, the ICCs of mathematics performance was 47% in Shanghai-China, and 22% in the United States, which indicated the necessity to use the multilevel modeling approach for both samples. Student OTL and mathematics anxiety, as well as student-level covariates, were included in Model 1. These variables explained 21% of the student-level variance and 42% of the school-level variance of mathematics performance in Shanghai-China, and 36% of the student-level variance and 44% of the school-level variance of mathematics performance in the United States. Student grade and ESCS showed significant effects on student mathematics performance in both Shanghai-China and United States. With higher relative grades and higher ESCS, students tended to achieve better performance controlling for other variables in both samples. Student gender did not show any significant effect on student mathematics performance in either Shanghai-China or United States. In both Shanghai-China and United States, OTL showed significant and positive effects on student mathematics performance (in Shanghai-China, β = 40.59,*p* < 0.001; in the United States, β = 48.33,*p* < 0.001), and mathematics anxiety showed significant and negative effects on student mathematics performance (in Shanghai-China, β = −36.54,*p* < 0.001; in the United States, β = −30.12,*p* < 0.001). This result indicated that students with more content exposure in mathematics and lower levels of mathematics anxiety might achieve better mathematics performance on average. Student problem-solving performance was then added to the model to examine its effect on student mathematics performance. Its effects were significantly positive in both samples (in Shanghai-China, β = 0.73,*p* < 0.001; in the United States, β = 0.70,*p* < 0.001), which explained an additional 38% of the student-level variance and 35% of the school-level variance of mathematics performance in Shanghai-China and 39% of the student-level variance and 20% of the school-level variance of mathematics performance in the United States. In other words, the higher problem-solving skills students had, the better mathematics performance they might get.

**TABLE 4:** Effects of OTL on student mathematics performance.

		Shanghai-China			United States	
		
	Null model	Model 1	Model 2	Null model	Model 1	Model 2
**Fixed effects**						
Intercept	612.66(1.03)***	619.89(19.13)***	224.75(39.55)***	486.70(1.42)***	448.94(10.63)***	97.32(25.38)***
Grade		27.87(4.63)***	8.39(3.71)*		25.24(3.76)***	8.39(3.01)**
Sex		5.36(3.67)	–13.91(3.39)***		2.68(3.88)	–0.36(3.20)
ESCS		9.06(2.82) **	1.40(2.30)		11.44(2.48)***	4.31(1.46)**
OTL		40.59(6.42)***	26.27(4.92)***		48.33(3.56)***	24.89(2.63)***
Mathematics anxiety		–36.54(2.92)***	–22.75(3.24)***		–30.12(2.66)***	–7.60(1.97) ***
Problem-solving performance			0.73(0.07) ***			0.70(0.05) ***
**Random effects**						
Student level σ^2^	5,280.86(240.42)***	4,189.47(181.46)***	2,182.78(399.88)***	6,078.58(274.07)***	3,880.62(180.05)***	1,528.88(316.72)***
School level τ_00_	4,613.59(286.42)***	2,696.85(268.33)***	1,080.05(123.51)***	1,740.33(167.78)***	980.80(118.60)***	626.85(64.19)***

*Standard errors are reported in parentheses.*

***p < 0.001.

**p < 0.01.

*p < 0.05.

### The Indirect Effects of OTL on Student Problem-Solving Performance and Mathematics Performance via Mathematics Anxiety

**FIGURE 2:**
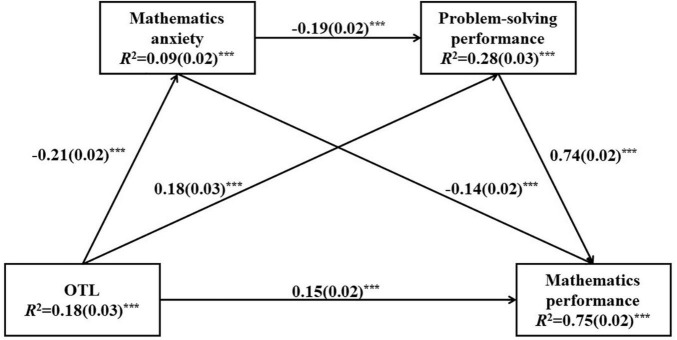
Sequential mediation model of opportunity to learn (OTL) as a predictor on student mathematics performance in Shanghai-China. Standardized path coefficients (Beta) are displayed for all paths. Standard errors are reported in parentheses. ***p < 0.001; **p < 0.01; *p < 0.05.

**FIGURE 3:**
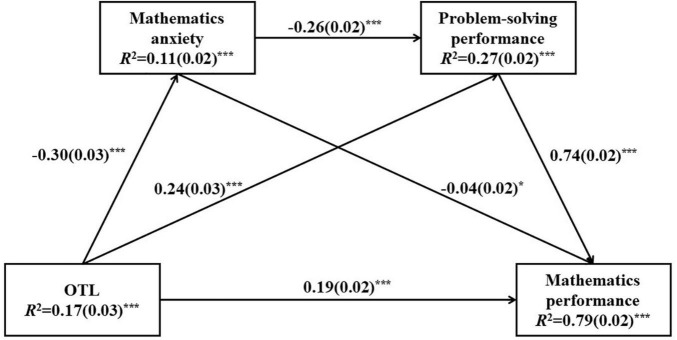
Sequential mediation model of opportunity to learn (OTL) as a predictor on student mathematics performance in United States. Standardized path coefficients (Beta) are displayed for all paths. Standard errors are reported in parentheses. ***p < 0.001; **p < 0.01; *p < 0.05.

The results from the multilevel models supported our hypotheses that OTL played significant influences on student mathematics anxiety, problem-solving performance, and mathematics performance. In addition, mathematics anxiety showed significant negative effects on student problem-solving performance and mathematics performance. To further examine the indirect effects of OTL on student problem-solving performance and mathematics performance *via* mathematics anxiety, saturated path models were built in both Shanghai-China and United States, after accounting for the effects of student gender, grade, and ESCS on OTL, mathematics anxiety, problem-solving performance, and mathematics performance. The results are presented in Figures 2, 3.

Student gender, grade, and ESCS explained about 18% of the variance in OTL in Shanghai-China and 17% of the variance in OTL in the United States. These covariates with OTL explained about 9% of the variance in mathematics anxiety in Shanghai-China and 11% of the variance in mathematics anxiety in the United States. About 28% of the variance in problem-solving performance in Shanghai-China and 27% of the variance in problem-solving performance in the United States were explained in the path model, and about 75% of the variance in mathematics performance in Shanghai-China and 79% of the variance in mathematics performance in the United States were explained.

First, the results showed significant effects of OTL on student mathematics anxiety in Shanghai-China and United States (in Shanghai-China, *standardized estimate* =–0.21, *p* < 0.001; in the United States, *standardized estimate* =–0.30, *p* < 0.001), suggesting that the more content coverage students were exposed to, the less anxiety they might feel when they dealt with mathematics. Compared to the United States sample, the direct effect of OTL on student mathematics anxiety was smaller in Shanghai-China. OTL also showed significant effects on student problem-solving performance (in Shanghai-China, *standardized estimate* = 0.18, *p* < 0.001; in the United States, *standardized estimate* = 0.24, *p* < 0.001) and mathematics performance (in Shanghai-China, *standardized estimate* = 0.15, *p* < 0.001; in the United States, *standardized estimate* = 0.19, *p* < 0.001). These results were consistent with the findings in multilevel models, that was to say, the more content coverage students had in mathematics, the better performance they might achieve in problem-solving and mathematics tests. Compared to the United States sample, the magnitudes of the direct effects of OTL on student problem-solving performance as well as on mathematics performance were smaller in Shanghai-China.

Second, significant and negative effects of mathematics anxiety were found on student problem-solving performance (in Shanghai-China, *standardized estimate* =–0.19, *p* < 0.001; in the United States, *standardized estimate* =–0.26, *p* < 0.001) and mathematics performance (in Shanghai-China, *standardized estimate* =–0.14, *p* < 0.001; in the United States, *standardized estimate* =–0.04, *p* < 0.05). The more anxiety student felt when they confronted mathematics, the lower performance they might get in problem-solving and mathematics tests. The direct effect of mathematics anxiety on student problem-solving performance was stronger in the United States than that in Shanghai-China, but mathematics anxiety showed a stronger direct effect on student mathematics performance in Shanghai-China. In addition, this path model also conformed to a significant and positive association between student problem-solving performance and mathematics performance (in Shanghai-China, *standardized estimate* = 0.74, *p* < 0.001; in the United States, *standardized estimate* = 0.74, *p* < 0.001).

The indirect effects of OTL on student problem-solving performance and mathematics performance were also calculated and tested for both samples (in Table 5). The indirect effects of OTL on student problem-solving performance *via* mathematics anxiety were statistically significant (in Shanghai-China, *standardized estimate* = 0.04, *p* < 0.001; in the United States, *standardized estimate* = 0.08, *p* < 0.001), suggesting that the OTL not only showed positively direct effects on student problem-solving performance, but also had a positive indirect effect on student problem-solving performance through lowering students’ mathematics anxiety. The more mathematics content coverage students had, the less likely they might feel anxious when they dealt with mathematics, and the better problem-solving performance they might get.

**TABLE 5:** Indirect effects of OTL on student mathematics performance.

Path	β(*SE*)	95% CI
**Shanghai-China**		
1. OTL→ Mathematics anxiety→ Problem-solving performance	0.04(0.01)***	[0.03, 0.05]
2. OTL→ Mathematics anxiety→ Mathematics performance	0.03(0.01)***	[0.02, 0.04]
3. OTL→ Problem-solving performance→ Mathematics performance	0.13(0.02)***	[0.09, 0.18]
4. OTL→ Mathematics anxiety→ Problem-solving performance→ Mathematics performance	0.03(0.01)***	[0.02, 0.04]
**United States**		
1. OTL→ Mathematics anxiety→ Problem-solving performance	0.08(0.01)***	[0.06, 0.10]
2. OTL→ Mathematics anxiety→ Mathematics performance	0.01(0.01)	[-0.00, 0.02]
3. OTL→ Problem-solving performance→ Mathematics performance	0.17(0.02)***	[0.13, 0.22]
4. OTL→ Mathematics anxiety→ Problem-solving performance→ Mathematics performance	0.06(0.01)***	[0.04, 0.07]

*Standardized results are displayed for all indirect effects. Standard errors are reported in parentheses.*

***p < 0.001.

**p < 0.01.

*p < 0.05.

The indirect effects of OTL on student performance mediated by problem-solving performance, and sequentially mediated by mathematics anxiety and problem-solving performance were significantly positive in both Shanghai-China and United States. In Shanghai-China, the standardized estimate of the indirect effect of OTL on mathematics performance mediated by problem-solving performance was 0.13 (*p* < 0.001), and the indirect effect of OTL on student mathematics performance *via* mathematics anxiety and problem-solving performance was 0.03 (*p* < 0.001). In the United States, the standardized estimates of the indirect effect of OTL on mathematics performance mediated by problem-solving performance, and sequentially mediated by mathematics anxiety and problem-solving performance were 0.17 and 0.06 (*p*s < 0.001). In both Shanghai-China and United States, the more content coverage students had in their daily mathematics lessons, the less anxious they might feel when they dealt with mathematics, the better problem-solving skills they might perform, and all of these might promote student mathematics performance. The indirect effect of OTL on student mathematics performance *via* mathematics anxiety was statistically significant in Shanghai-China (*standardized estimate* = 0.03, *p* < 0.001), but not significant in the United States (*standardized estimate* = 0.01, *p* > 0.05).

## Discussion

The instructional interventions on student mathematics anxiety and mathematics performance are of great interest for both researchers and educators. Overall, the findings in the current study supported our hypotheses that OTL, measured as the content coverage in mathematics, had not only significant direct effects on student problem-solving performance and mathematics performance, but also indirect effects on them mediated by student mathematics anxiety in Shanghai-China and United States samples controlling for student gender, grade, and ESCS. In addition to the direct effects, one mechanism by which OTL affected student mathematics-related outcomes was through affecting students’ achievement emotions. By getting more OTL or more content coverage in daily mathematics lessons, students may feel more in control and less anxious when they are doing mathematics, which may benefit student mathematics-related outcomes.

The result firstly showed a negative relationship between mathematics anxiety and student problem-solving performance and mathematics performance controlling for the effects of student gender, grade, ESCS, and OTL. As discussed in the introduction, mathematics anxiety is identified as one of the important risk factors for student success in mathematics. A negative and moderate correlation between mathematics anxiety and student problem-solving performance/mathematics performance was found in both Shanghai-China and the United States in the current study, which was consistent with the literature (Barroso et al., 2021). The more worried students feel about the difficulties of the mathematics classes for them, the tenser they get when they have to do mathematics homework, the more nervous they feel when doing mathematics problems, the more helpless they feel doing a mathematics problem, and the more they worry about their poor mathematics grades (OECD, 2014d), the lower problem-solving performance and mathematics performance they may get.

In the multilevel models, when the effects of problem-solving performance on student mathematics performance were taken into consideration, the effects of mathematics anxiety on student mathematics performance dropped down in both Shanghai-China and United States. One possible explanation for this result is that mathematics anxiety may play an effect on student mathematics performance by lowering their use of mathematics-related problem-solving skills. The results from the path models provide some evidence for it, whereas the relative effect of mathematics anxiety on problem-solving performance was a little bit larger than that on mathematics performance, and the indirect effect of mathematics anxiety on student mathematics performance mediated by problem-solving performance was statistically significant, no matter which sample was used. Ramirez et al. (2016) found a similar result that student mathematics anxiety was negatively related to their problem-solving strategies, which in turn had an effect on student mathematics performance. They suggested that students with higher mathematics anxiety would avoid using advanced problem-solving skills, which lead to lower performance in mathematics. It is worth noticing that the relative effects of mathematics anxiety on problem-solving performance and of problem-solving performance on mathematics performance were similar in the Shanghai-China and United States samples in the current study, but the relative effects of mathematics anxiety on student mathematics performance differed in the path models. The multilevel models provided a similar result. A larger decline of mathematics anxiety effect on student mathematics performance was found in the United States after the problem-solving performance was included in the model. Consistent with previous studies (Lee, 2009; Morony et al., 2013), in the current study, students from Shanghai-China reported higher mathematics anxiety in spite of their higher scores on problem-solving performance and mathematics performance. Mathematics anxiety demonstrated both direct and indirect effects on student mathematics performance in Shanghai-China, but only indirect effects *via* problem-solving performance in the United States. This result may reflect the cultural differences in the mechanisms by which mathematics anxiety affects student mathematics performance.

As expected, OTL showed significant effects on student mathematics anxiety, problem-solving performance, and mathematics performance. In the literature, OTL is an important instructional factor. Although various measures of OTL are used in the previous studies, a consistently positive and significant effect of OTL on student mathematics performance is documented (Cogan et al., 2001; Byrnes and Miller, 2007; Lleras, 2008; Byrnes and Wasik, 2009; Lee and Chen, 2009; Reeves, 2012; Albano and Rodriguez, 2013; Grouws et al., 2013; Tar et al., 2013; Ottmar et al., 2014; Schmidt et al., 2015; Santibanez and Fagioli, 2016). In the current study, the OTL is measured as the content coverage of different key mathematics topics in daily mathematics lessons. Considering the fixed length of schooling years, and constant time for mathematics instructions in a school year, when measuring OTL on the content coverage scale with a list of necessary, important, and advanced mathematics topics across years, this indicator of OTL, in fact, reflects the content exposure and emphases of important topics in students’ daily mathematics lessons. The positive effects of OTL on student mathematics performance and problem-solving performance were found in both Shanghai-China and the United States in the current study. The better OTL students have in their mathematics lessons, the higher performance they may achieve in mathematics and mathematics-related cognitive skills. The magnitudes of the effects of OTL on student problem-solving performance and mathematics performance were similar in Shanghai-China and the United States.

In terms of the effect of OTL on student mathematics anxiety, a significant and negative effect was found in both the Shanghai-China and United States samples. OTL showed similar impacts on student mathematics anxiety in both samples. That was to say, better content coverage in daily mathematics lessons may also make students feel less anxious in the mathematics-related situations. According to the control-value theory, by providing students with a higher instructional quality in mathematics, students may feel more confident and have a higher sense of control over the mathematics-related situations, which may lead to a lower level of anxiety (Pekrun et al., 2002; Pekrun, 2006). Previous research showed the beneficial effects of instructional quality on student mathematics anxiety by examining the effects of off-class tutoring or training programs, which provided extra learning opportunities in mathematics content and skills (Supekar et al., 2015; Passolunghi et al., 2020). In the current study, OTL is an index of the cumulated content coverage of mathematics topics in daily mathematics instructions. It may provide some ideas on alleviating students’ mathematics anxiety by daily instructions in mathematics, in addition to the off-class remediation programs.

The hypothesized indirect effect of OTL on student problem-solving performance *via* mathematics anxiety was supported in the current study. The indirect effect of OTL on student mathematics performance sequentially mediated by mathematics anxiety and problem-solving performance was significant in both samples. These mechanisms for the indirect effects of OTL may be explained by the control-value theory (Pekrun et al., 2002; Pekrun, 2006) and attentional control theory (Eysenck and Calvo, 1992; Eysenck et al., 2007; Eysenck and Derakshan, 2011). The control-value theory (Pekrun et al., 2002; Pekrun, 2006) pays attention to the antecedents of achievement emotions, and mathematics anxiety is often discussed. Instructional quality is listed as one important antecedent for mathematics anxiety. Rather than the antecedents, attentional control theory focuses on the mechanisms by which mathematics anxiety affects student performance. The attentional control theory suggests that students may not have enough cognitive resources in using higher cognitive strategies or skills when they are under anxiety. Based on these two theories, it is not surprising that OTL showed direct effects on student problem-solving performance and mathematics performance, as well as indirect effects mediated by mathematics anxiety in the current study. Although limited empirical research has explored the direct and indirect effects of instructional quality on student mathematics performance through mathematics anxiety, a large amount of research confirmed the positive effects of qualified mathematics content on student mathematics-related cognitive skills and mathematics performance (Cogan et al., 2001; Byrnes and Miller, 2007; Lleras, 2008; Byrnes and Wasik, 2009; Lee and Chen, 2009; Reeves, 2012; Albano and Rodriguez, 2013; Grouws et al., 2013; Tar et al., 2013; Ottmar et al., 2014; Schmidt et al., 2015; Santibanez and Fagioli, 2016), as well as the studies on training or tutoring programs provided some evidence for the effects of mathematics content and skills on student mathematics anxiety (Frenzel et al., 2007; Supekar et al., 2015; Ching et al., 2020; Passolunghi et al., 2020; Tomasetto et al., 2021). When students get better content coverage on the key topics in their daily mathematics lessons, they may feel less anxious about mathematics and they may be capable to use proper cognitive skills or strategies to solve complex problems, and all of these may lead to a better mathematics performance. The role of the mathematics content coverage may not be only important for improving student performance, but also significant for alleviating students’ mathematics anxiety. In the current study, another hypothesized indirect effect of OTL on student mathematics performance *via* mathematics anxiety was only found in Shanghai-China, but not in the United States. As discussed above, the effect of mathematics anxiety on student mathematics performance was small in size when student problem-solving performance was considered in the United States. Compared to the Shanghai-China sample, this result may suggest that in the United States sample, mathematics anxiety affects student mathematics performance mainly through reducing their use of problem-solving skills. The cultural differences in the structures of mathematics anxiety and the allocations of attention and cognitive resources (Lee, 2009; Morony et al., 2013; Zhu and Meyer, 2020) when students are under high-anxious situations may provide another plausible explanation for this result.

Mathematics anxiety attracts large attention in both research and practices as it is widely found in different schooling systems, and shows adverse effects on student mathematics performance and their future success in mathematics. The current study illustrated the direct and indirect effects of OTL in mathematics with a measure describing the content coverage in mathematics, on student mathematics performance *via* mathematics anxiety. The high-quality OTL students get in their daily mathematics lessons may not only bring direct benefits to their mathematics performance and mathematics-related problem-solving skills, but also help with students’ mathematics anxiety. The direct and indirect effects of OTL were found in both eastern and western samples. The school-level intervention on daily mathematics instructions may be more efficient and practical. Thus, mathematics curriculum designers, instructors, and interventions aiming at promoting students’ success in mathematics, as well as the educators and programs aiming at lowering students’ mathematics anxiety should pay a close attention to what students learn in their daily mathematics lessons.

The OTL in mathematics in the current study describes the mathematics content coverage reported by students, which measures OTL just from students’ exposure view, and it is only one aspect of mathematics instruction. Previous studies used different OTL measures, like different mathematics curricula (Grouws et al., 2013) and percentage of textbook lessons taught (Tar et al., 2013), and examined their effects on student mathematics anxiety, mathematics performance, and problem-solving performance. The OTL is measured as the content coverage in mathematics in the current study. Its results may not be directly comparable with the research using different OTL measures. Furthermore, the control-value theory indicates that the teaching and learning environment, as well as teachers’ instructional practices and behaviors, will have some effects on student mathematics anxiety (Pekrun et al., 2002; Pekrun, 2006). They, at the same time, may also show impacts on the development of students’ mathematics-related cognitive skills (Lee and Chen, 2009). Further efforts should be given to exploring the effects of different aspects of daily instructional practices on student mathematics anxiety and cognitive skills, in addition to the mathematical remediation programs or off-class projects. The current study used the dataset from PISA 2012 for its high quality in mathematics and problem-solving tests. For the limited length in the student contextual questionnaire, only five items were used to measure student mathematics anxiety. In future studies, mathematics anxiety should be measured with more precision. PISA 2012 used a random rotated block design for the student survey. Only one-third of students answered OTL and mathematics anxiety items, which left about 10 students in each school. Further research consideration should be given to the generalization of the current results. In addition, the environmental factors and student characteristics, like teacher quality and educational resources, and student self-efficacy and mathematical interests, are highly likely to be correlated with OTL, student mathematics anxiety, and mathematics outcomes. For the limited measure on these variables in the PISA survey, they were not included in the current study, and need considerations in future research. Last but not least, a longitudinal study will help to identify the reciprocal relationships between mathematics anxiety, problem-solving skills, and mathematics performance, as well as the effects of OTL on them. Readers should be careful when generalizing the current results, and additional research should be conducted to understand the direct and indirect effects of instructional quality in mathematics on student mathematics performance and mathematics-related cognitive skills *via* mathematics anxiety.

## Data Availability Statement

Publicly available datasets were analyzed in this study. This data can be found here: http://www.oecd.org/pisa/data/.

## Author Contributions

SG designed the study and took a leading role in writing the manuscript. SL contributed to the literature review and data analyses. Both authors contributed to the article and approved the submitted version.

## Conflict of Interest

The authors declare that the research was conducted in the absence of any commercial or financial relationships that could be construed as a potential conflict of interest.

## Publisher’s Note

All claims expressed in this article are solely those of the authors and do not necessarily represent those of their affiliated organizations, or those of the publisher, the editors and the reviewers. Any product that may be evaluated in this article, or claim that may be made by its manufacturer, is not guaranteed or endorsed by the publisher.
